# The Endosymbiont *Arsenophonus* Is Widespread in Soybean Aphid, *Aphis glycines*, but Does Not Provide Protection from Parasitoids or a Fungal Pathogen

**DOI:** 10.1371/journal.pone.0062145

**Published:** 2013-04-16

**Authors:** Jason A. Wulff, Karrie A. Buckman, Kongming Wu, George E. Heimpel, Jennifer A. White

**Affiliations:** 1 Department of Entomology, University of Kentucky, Lexington, Kentucky, United States of America; 2 USDA, Agricultural Research Service, Center for Grain and Animal Health Research Unit, Manhattan, Kansas, United States of America; 3 Department of Entomology, University of Minnesota, St. Paul, Minnesota, United States of America; 4 State Key Laboratory for Plant Diseases and Insect Pests, Institute of Plant Protection, Chinese Academy of Agricultural Sciences, Beijing, China; International Atomic Energy Agency, Austria

## Abstract

Aphids commonly harbor bacterial facultative symbionts that have a variety of effects upon their aphid hosts, including defense against hymenopteran parasitoids and fungal pathogens. The soybean aphid, *Aphis glycines* Matsumura (Hemiptera: Aphididae), is infected with the symbiont *Arsenophonus* sp., which has an unknown role in its aphid host. Our research goals were to document the infection frequency and diversity of the symbiont in field-collected soybean aphids, and to determine whether *Arsenophonus* is defending soybean aphid against natural enemies. We performed diagnostic PCR and sequenced four *Arsenophonus* genes in soybean aphids from their native and introduced range to estimate infection frequency and genetic diversity, and found that *Arsenophonus* infection is highly prevalent and genetically uniform. To evaluate the defensive role of *Arsenophonus*, we cured two aphid genotypes of their natural *Arsenophonus* infection through ampicillin microinjection, resulting in infected and uninfected isolines within the same genetic background. These isolines were subjected to parasitoid assays using a recently introduced biological control agent, *Binodoxys communis* [Braconidae], a naturally recruited parasitoid, *Aphelinus certus* [Aphelinidae], and a commercially available biological control agent, *Aphidius colemani* [Braconidae]. We also assayed the effect of the common aphid fungal pathogen, *Pandora neoaphidis* (Remaudiere & Hennebert) Humber (Entomophthorales: Entomophthoraceae), on the same aphid isolines. We did not find differences in successful parasitism for any of the parasitoid species, nor did we find differences in *P. neoaphidis* infection between our treatments. Our conclusion is that *Arsenophonus* does not defend its soybean aphid host against these major parasitoid and fungal natural enemies.

## Introduction

Maternally inherited bacterial endosymbionts are common in arthropods [1]–[5]. Many insects are infected with obligate nutritional endosymbionts that are required for survival, e.g. *Buchnera aphidicola* in aphids [1], [2], [6]. In contrast, facultative endosymbionts are not strictly required for insect survival, but can provide a selective advantage in certain ecological contexts [7]. For example, facultative endosymbionts have been shown to provide their hosts with heat shock resistance [8], modify host color [9], and potentially facilitate host plant colonization [10]. A subset of these facultative endosymbionts can also defend their insect hosts against natural enemies such as parasitoids, entomopathogenic fungi, viruses, and nematodes [11]–[14].

Bacterial symbionts in the genus *Arsenophonus* are estimated to infect approximately 5% of arthropods [4], [15]. In the parasitoid wasp *Nasonia vitripennis, Arsenophonus nasoniae* acts as a male killing reproductive parasite [16]–[19]. Other strains are thought to be obligate symbionts of triatomine bugs, hippoboscid and streblid flies, and lice [20]–[22], and yet others are plant pathogens [23]–[25]. *Arsenophonus* is also found in multiple whitefly, psyllid, and aphid species [26]–[30], but its function among these hosts remains uncharacterized. However, there have been suggestions that *Arsenophonus* may play a defensive role. In a geographic survey of the lerp psyllid, *Glycaspis brimblecombei,* Hansen et al. (2007) found a positive correlation between parasitism and the frequency of *Arsenophonus* infection, potentially indicating that *Arsenophonus* provides the psyllid with a selective advantage in populations under heavy parasitism pressure [31].

If *Arsenophonus* provides defense against natural enemies, then it could be an important consideration in biological control programs against *Arsenophonus-*bearing pests. For example, a defensive symbiont that is present at low prevalence within a population could become common under selective pressure provided by a newly released classical biological control agent, thus undercutting the efficacy of the agent [32], [33]. Alternatively, laboratory populations, which experience vastly different selective environments and frequent population bottlenecks [34], might be expected to have a different frequency of symbiont infection than field populations. In such a case, conclusions about natural enemy efficacy drawn from laboratory studies may have little bearing on natural enemy performance in the field.

Multiple important pest species are infected with *Arsenophonus*, including the lerp psyllid, the cotton aphid, *Aphis gossypii,* the sweet potato whitefly, *Bemisia tabaci,* and the soybean aphid, *Aphis glycines* [26], [31], [35], [36]. Soybean aphid is a serious invasive pest of soybeans in North Central United States, causing extensive yield loss and requiring intensive pesticide applications to a crop that required little pesticide input prior to the introduction of the soybean aphid [37]. Early parasitism surveys in North America found that soybean aphids were infrequently parasitized [38]–[40], leading to ongoing biological control investigations that incorporate augmentation of ambient fungal pathogens and introduction of parasitoids from the aphid's native range [41]–[43]. The function and prevalence of *Arsenophonus* in field populations of soybean aphid has the potential to affect these pest management tactics.

The goals of this study were 1) to document the frequency and diversity of *Arsenophonus* infection in field-collected soybean aphids from the aphids' native and introduced range and 2) to investigate whether *Arsenophonus* protects soybean aphid against parasitoid wasps or entomopathogenic fungi by assessing natural enemy efficacy against infected versus experimentally cured aphid isolines. For the former goal, we performed *Arsenophonus* diagnostic PCR on six native and seven introduced populations of soybean aphid, followed by multi-locus strain typing (MLST) of *Arsenophonus* using 3 bacterial genes [30], [44]. For the latter goal, we assayed three species of parasitoid wasp and one species of fungal pathogen. The first parasitoid species assayed was *Binodoxys communis*, which currently is the only exotic parasitoid to have been intentionally released in the United States to control the soybean aphid as part of a classical biological control program [42]. The second wasp, *Aphelinus certus*, has been identified from parasitized North American soybean aphids, although estimates of parasitism rates are still forthcoming. This parasitoid is native to China, was potentially co-introduced with soybean aphid, and is of interest as a biological control agent [45]. The third wasp, *Aphidius colemani,* is a commercially-available generalist parasitoid of aphids that is known to be susceptible to a defensive symbiont in pea aphid [46]. The aphid fungal pathogen, *Pandora neoaphidis*, is also known to be susceptible to defensive symbionts in pea aphid, and is being investigated for augmentative biological control of the soybean aphid [12], [47], [48].

## Results

### Geographic survey

When the prevalence of *Arsenophonus* in native and introduced populations of the soybean aphid was surveyed, we found that the symbiont was very common in all examined populations (Table 1). In the introduced North American range, a mean (±S.E.) of 98±1% of aphids were infected, which was slightly, but significantly, higher than the 85±6% infection found in the native Asian range (Wald = 2.128, df = 11, P = 0.0334).

**Table 1:** Soybean aphid, *Aphis glycines,* collection locations, year collected, collector, and *Arsenophonus* prevalence.

*Locality*	*Year*	*Collector*	*Arsenophonus positive/Aphids screened*
Native			
Hebei Province, China	2008	Wu Kongming	8/8
Shangdong Province, China	2008	Wu Kongming	9/10
Guangxi Province, China	2008	Wu Kongming	10/10
Hangzou District, China	2008	Wu Kongming	7/10
Yangling District, China	2008	Wu Kongming	9/10
Harbin Province, China	2008	Wu Kongming	5/8
Introduced			
Whitley Co., Indiana, USA	2008	Marc Rhainds	23/25
Tippecanoe Co., Indiana, USA	2008	Marc Rhainds	10/10
Wabash Co., Indiana, USA	2008	Marc Rhainds	5/5
Huntington Co., Indiana, USA	2008	Marc Rhainds	5/5
Olmsted Co., Minnesota, USA	2008	Fritz Breitenbach	5/5
Waseca Co., Minnesota, USA	2008	George Heimpel	5/5
Fayette Co., Kentucky, USA	2011	Jason Wulff	27/28

### Arsenophonus MLST

*Arsenophonus fbaA, ftsK, yaeT* genes were sequenced from one aphid from each of our surveyed populations [30], [44]. We did not detect any genetic variation among sequences from the native and introduced populations, giving no evidence for multiple strains of *Arsenophonus* within soybean aphid.

### Parasitism assays

**Figure 1:**
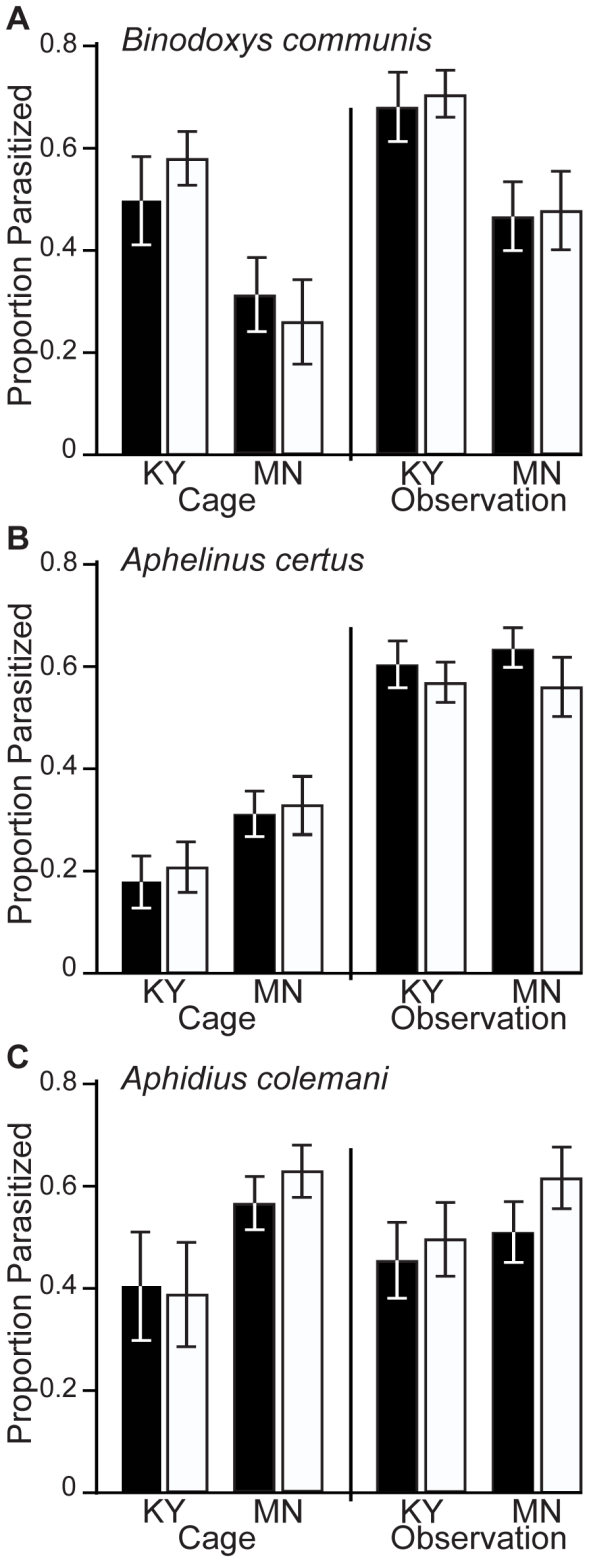
Mean (±SE) proportion of soybean aphids parasitized by *Binodoxys communis* (A), *Aphelinus certus* (B), and *Aphidius colemani* (C). Black bars represent naturally *Arsenophonus*-infected soybean aphids and white bars represent experimentally cured isolines with the same genetic background. Two isoline pairs (KY and MN) were each evaluated in two experiments (cage and observation assays) for each parasitoid species. No significant differences were detected in any assay.

The influence of *Arsenophonus* on soybean aphid susceptibility to parasitism was assessed using three different parasitoids. Parasitism by the introduced biological control agent *B. communis* did not differ significantly between *Arsenophonus-*infected and experimentally cured aphids of a Kentucky (KY) origin isoline within either a cage assay (t = 0.88, df = 18, P = 0.39), or an observation assay (t = 0.22, df = 22, P = 0.83; Figure 1A). Parasitism of a Minnesota (MN) origin isoline of aphids was substantially lower than the KY isoline, but again did not differ between *Arsenophonus-*infected and experimentally cured aphids in either the cage assay (t = 0.86, df = 22, P = 0.40), or the observation assay (t = 0.12, df = 22, P = 0.90).

There were no differences in *A. certus* parasitism of the KY isoline in the cage assay (t = 0.38, df = 22, P = 0.71) or the observation assay (t = 0.52, df = 20, P = 0.61), nor of the MN isoline in the cage assay (t = 0.02, df = 19, P = 0.98) or the observation assay (t = 0.99, df = 18, P = 0.33; Figure 1B). *A. certus* had the greatest disparity in performance between the two assays, with very low rates of parasitism for cage assays compared to the observation assays.

There were also no differences in proportion parasitism by *A. colemani* between infected and experimentally cured soybean aphid for either isoline or parasitism assay (KY cage assay: t = 0.33, df = 20, P = 0.75; KY observation assay: t = 0.29, df = 24, P = 0.77; MN cage assay: t = 0.97, df = 20, P = 0.34; MN observation assay: t = 1.87, df = 18, P = 0.07; Figure 1C).

### Fungal assays

In a challenge using the entomopathogenic fungus *P. neoaphidis*, observed proportions of infection were highly variable, ranging from 0 to 0.76 per replicate. Mean (± SE) proportion *P. neoaphidis* infection in the *Arsenophonus* infected and uninfected aphids in the KY isoline were 0.15±0.05 and 0.12±0.06 respectively, and arcsine squareroot transformed values did not differ significantly from one another (t = 0.58, df = 18, P = 0.57). Likewise, *Arsenophonus* infected and uninfected aphids in the MN isoline had 0.22±0.07 and 0.13±0.05 proportion infected, and again did not differ significantly from one another (t = 1.46, df = 18, P = 0.16).

## Discussion

Our primary goal was to assess whether *Arsenophonus* defends soybean aphid against natural enemies. Using three parasitoid wasp species, we found no evidence that *Arsenophonus* provides this defense in either of two genotypes of soybean aphid. All three species of parasitoids were able to successfully attack soybean aphid, and there were no significant differences in successful parasitism of *Arsenophonus-*infected versus cured aphids in either cage or observation assays. Likewise, we found no difference in aphid mortality from the fungus *P. neoaphidis* based on *Arsenophonus* infection.

Our aggregated results indicate that *Arsenophonus* is likely not a defensive symbiont in soybean aphid, but some caveats should be considered. First, we used only two genotypes of aphids, which were infected with the same strain type of *Arsenophonus*, based on identical *Arsenophonus* ribosomal and MLST sequences. It is possible that other *Arsenophonus* strains may provide protection to other genotypes of soybean aphid host. For example, different strains of the bacterial endosymbiont *Hamiltonella defensa* provide differential protection against parasitism to pea aphid based on the presence or absence and type of APSE phage [49]. Additionally, a strain of *Regiella insecticola* was recently shown to protect its aphid host against parasitism, a trait not previously associated with the symbiont [46], indicating that bacterial strains can vary in their defensive properties. However, in soybean aphid our broad MLST survey of *Arsenophonus* did not identify any additional bacterial strains in either the native or introduced range, indicating that hypothetical alternate strain types are rare, if they exist at all. Furthermore, soybean aphid is a recent introduction to North America, and is notably lacking in genetic diversity [50]; consequently, it seems unlikely that additional sampling of aphid/symbiont genotypes in the invaded range would yield different results.

We limited our parasitism assays to wasp species relevant to the North American introduced range of soybean aphid. *B. communis* and *A. certus* are both of interest for biological control and represent two different families of parasitoids (Braconidae and Aphelinidae, respectively), the latter being a more generalized parasitoid species [45], [51]. However, there is growing evidence that defensive symbiont-mediated selection can favor parasitoid genotypes that are insensitive to the symbiont [52]. The high prevalence of *Arsenophonus* infection in the field makes it likely that field-collected parasitoids of soybean aphid have encountered and potentially adapted to the symbiont. *A. colemani,* the third wasp we assayed, was commercially cultured on other aphid species and presumably naïve to soybean aphid, yet it was also unaffected by *Arsenophonus*.

Although our results indicate that *Arsenophonus* does not defend its host against these natural enemies, it does have a very high infection rate in both the introduced and native populations. Several possible explanations could underlie this widespread infection. First, *Arsenophonus* could manipulate host reproduction. Reproductive manipulation is a common means by which endosymbionts promote their own infection, and has recently been documented in the sexual generation of pea aphid by the endosymbiont *Spiroplasma* [53], [54]. Second, *Arsenophonus* could be providing other context-specific benefits to soybean aphid, e.g. heat tolerance, defense against other pathogens [8], [14], or general fecundity or longevity effects [55]. Third, *Arsenophonus* may be transmitted horizontally, either directly between aphids or indirectly through the plant [56], [57]. Finally, high fidelity vertical transmission, coupled with a very low metabolic cost to the host, could permit *Arsenophonus* to persist in a population without any benefit to the host [58]. However, other endosymbionts that had been considered previously to be neutral passengers were subsequently found to be extremely beneficial to their hosts under certain circumstances [13], [59]. Given the very high prevalence of *Arsenophonus* in soybean aphid, it is therefore reasonable to presume that *Arsenophonus*, too, provides soybean aphid with a context-specific benefit that remains to be elucidated.

## Materials and Methods

### Geographic survey

To evaluate the prevalence of *Arsenophonus*, soybean aphids were collected from the Asian native range and North American invasive range. Collections were made either at university agricultural stations or on private lands with landowner permission (Table 1). For each population, 30 adult aphids were collected from plants at least 1 meter apart to minimize sampling of siblings, and immediately placed in 95% ethanol. Five aphids were selected at random from each introduced range population and ten aphids were selected from each native range population for molecular analysis. We extracted DNA by homogenizing individual aphids in 100 µl of 10% w/v Chelex (Sigma-Aldrich, St Louis, MO, USA) in PCR-grade purified water. We added 6 µl of proteinase K to each sample, vortexed, incubated overnight at 56°C, and then incubated samples at 96°C for ten minutes. We screened for the presence of *Arsenophonus* using a diagnostic PCR protocol modified from Thao and Baumann [26], which uses *Arsenophonus* specific primers to amplify the intervening region between 16S and 23S rDNA: Ars23S-1 (5′-CGTTTGATG ATTCATAGTCAAA-3′) and Ars23S-2 (5′-GGTCCTCCAGTTAGTGTTACCCAAC -3′). Reactions totaled 10 µl, containing: 2.0 µl of DNA template, 1.0 µl of 25 mM MgCl_2_, 1.0 µl of 10 mM dNTP mixture, 1.0 µl of Invitrogen 10× buffer (MgCl_2_ free), 0.8 µl of 5.0 pmole µl^−1^ of each primer, 0.1 µl of 5 U/ µl Invitrogen Taq polymerase, and ddH_2_O to 10 µl. PCR conditions were: initial denature at 95°C for 5 min; followed by 30 cycles of (95°C, 30 s; 55°C, 30 s; 72°C, 45 s); and final elongation at 70°C for 10 min. All PCRs included negative and positive controls. Product from multiple samples was sequenced to confirm *Arsenophonus*. All sequences were identical and the shared sequence was submitted to Genbank (Accession number KC019882). As a further control of extraction quality, we ran samples with the primers CAIF (5′-GCCTGATGCAGCCATGCCGCGTGTATG-3′) and CAIR (5′-GTCATCCCCACCTTCC-3′) with the same PCR conditions as previously listed. These primers were developed by Dale et al. [60] to target *Arsenophonus* 16S sequence in the hippoboscid fly, *Pseudolynchia canariensis*. However, they reliably detected 16S sequence from the obligate symbiont *Buchnera aphidicola* in soybean aphid, as confirmed by sequencing results (Accession number KC019881). Because this obligate symbiont should be present in all extractions, any samples that failed to amplify *B. aphidicola* were considered to be of poor quality and discarded. To compare *Arsenophonus* infection prevalence between the native and introduced ranges, we used logistic regression (Arc v. 1.06). To avoid overrepresentation of heavily sampled geographic regions, aphids collected from within the same county were considered to come from a single population, and pooled prior to statistical analysis.

### MLST

We investigated potential genetic diversity in *Arsenophonus* using an MLST approach. We randomly selected a single extraction from each native and introduced population (Table 1), as well as from our two experimental colonies (KY and MN). We amplified DNA from each sample with the following primer sets: fbaAf (5′-GCYGCYAAAGTTCRTTCCC-3′) and fbaAr2 (5′-GGCAAATTAAATTTCTGCGCAACG-3′), ftsKf (5′-GTTGTYATGGTYGATGAATTTGC-3′) and ftsKr (5′-GCTCTTCATCACYTCAWAACC-3′), yaeTf (5′-GCATACGGTTCAGACGGGTTTG-3′) and yaeTr (5′-GCCGAAACGCCTTCAGA AAAG-3′).The PCR reaction recipe followed the protocol above and PCR conditions were: initial denature at 93°C for 3 min; 30 cycles of (93°C, 30 s; 52°C, 30 s; 72°C, 1 min); and final elongation at 72°C for 5 min [30], [44]. Because sequences generated from each population were identical for each of the genes, *fbaA*, *ftsK*, and *yaeT*, a single sequence per gene was submitted to Genbank (KC701199, KC701198, KC701197).

### *Arsenophonus* curing and colony maintenance

We used two soybean aphid clones for experimental manipulations. These clones were collected independently of the geographic survey specimens. One aphid clone, "KY", was initially collected in Fayette County, KY in 2009.The second clone, "MN", was originally collected in Ramsey County, MN and was maintained in culture at the University of Minnesota prior to transfer to Kentucky in 2010 (USDA Permit # P526P-10-00818). In addition to *Arsenophonus*, each aphid clone was screened diagnostically for other known bacterial symbionts of aphids [29], and examined for total bacterial diversity using denaturing gradient gel electrophoresis (DGGE) of bacterial 16S sequences [61]. The only bacterial endosymbionts detected were *Arsenophonus* and *Buchnera* (J. Wulff, unpublished data).

We cured these aphid clones of *Arsenophonus* infection using antibiotic microinjection, following a protocol modified from Oliver et al. [11]. Individual aphids from each clone were immobilized on a screen-covered pipette tip attached to vacuum, under a stereo microscope. Antibiotic was fed into a borosilicate microinjection needle attached to a syringe via tubing. Fourth-instar aphids were injected with 1.0 mg ml^−1^ ampicillin solution [62]. *Arsenophonus* is susceptible to ampicillin, but the aphid's primary symbiont, *Buchnera aphidicola*, is not [63]. After the initial injection, aphids were individually placed on excised soybean leaves maintained on 1% w/v agar, monitored for survivors, and a subset of offspring were checked for *Arsenophonus* via diagnostic PCR. This procedure was repeated for two subsequent generations using offspring of survivors from the previous bout of injections. Cured and infected isoline colonies were kept at 25± 1°C and 16L:8D on Asgrow® G4303 variety commercial soybeans in 10 cm pots. Plants were individually caged in 3.78 liter plastic jars that had panels of mesh to allow ventilation while preventing aphid escape. Aphids were transferred to new plants as needed, approximately twice per month, to avoid overcrowding and prevent alate production. All aphid isolines were maintained in this manner for at least 3 months prior to experiments. Five individuals from each soybean aphid isoline were screened with diagnostic PCR at least every 2 months to assure that the isoline retained the expected infection status. The cured aphid isolines never tested positive for *Arsenophonus*.

### Parasitism assays

We evaluated the influence of *Arsenophonus* in soybean aphid on parasitism success by three parasitoid wasp species. The classical biological control agent *Binodoxys communis* was initially collected in August 2002 near Harbin, in the Chinese province of Heilongjaing, and was maintained in quarantine in St. Paul, Minnesota prior to initiation of our colony in Kentucky (USDA-APHIS permit P526P-10-01532) [64]. *Aphelinus certus* was collected locally in Lexington, KY in August 2010 from parasitized soybean aphids. *Aphidius colemani* is a commercially available biological control agent of aphids (APHIPAR, Koppert Biological Systems, The Netherlands). Each species of parasitoid was maintained in culture with *Arsenophonus*-cured soybean aphids and soybean plants at 25 ±1°C and 16L:8D in the previously described culture jars with supplemental honey and water for at least two generations prior to use in parasitism assays.

### Cage parasitism assays

We conducted cage parasitism assays using methodology adapted from Oliver et al. [11]. For each *Arsenophonus* infected/cured isoline pair, we assayed parasitism success by each of the three parasitoid species in separate experiments (6 assays total). For each assay, 12 vegetative stage 2 (V2) soybean plants were infested with *Arsenophonus*-infected aphids and 12 V2 soybean plants were infested with *Arsenophonus*-cured aphids. We transferred a leaf with >100 juvenile aphids to each experimental plant. Experimental plants were covered with cup cages, constructed from 947 ml translucent plastic containers, organza screening material, and weather stripping to provide a tight seal between cage and pot. After allowing 24 h for aphid establishment, we culled the aphids to either 30 aphids (*A. certus* assays), or 50 aphids (*B. communis* and *A. colemani* assays). *B. communis* and *A. certus* assays were conducted primarily with 2^nd^ and 3^rd^ instar aphids, whereas *A. colemani* assays were conducted primarily with 3^rd^ and 4^th^ instar aphids [64], [65]. A single mated female wasp was introduced to each cup cage and removed after 24 h. If the wasp was dead or missing after this interval, the replicate was discarded. After 10 d, parasitized aphids (mummies) were counted, and proportion parasitism was calculated by dividing the number of mummies observed by the initial aphid number for that replicate. For each assay, the effect of aphid infection status on proportion parasitism was assessed using a t-test (IBM SPSS v20). Proportion data required an arcsine square-root transformation to satisfy the assumptions of the model.

### Observation assays

Six observation assays were conducted in parallel to the cage assays, using the same three parasitoid species and two aphid genotypes. For each experiment, soybean leaves infested with either *Arsenophonus*-infected or cured aphids of all instars were embedded, adaxial side, in 1% agar in 100×15 mm petri dishes. Five to ten wasps of the same species were aspirated onto the embedded leaf. Wasps were allowed to settle and then culled to four actively parasitizing wasps. Wasps were observed continuously under a dissecting microscope. When oviposition was observed, each stung aphid was moved to a 35 mm leaf disk embedded in 1% agar, until a total of 10–15 aphids were parasitized, constituting a replicate. This procedure was repeated with fresh wasps until 10 replicates were generated per treatment per assay.

We regularly removed aphid progeny from leaf disks to avoid confusing progeny with the original stung aphids. Wasp mummies typically formed within 5–7 days, regardless of the parasitoid species. On day 10, we calculated proportion parasitism by dividing the number of mummies by the number of aphids that had survived until just prior to mummy formation. Aphids that died prior to day 5 were excluded from the data. Proportions were arcsine square-root transformed and analyzed using a t-test for each assay.

### Fungal assays

To assess the effect of *Arsenophonus* infection status on soybean aphid susceptibility to the entomophthoralean fungus *P. neoaphidis*, we conducted bioassays of *Arsenophonus*-infected versus cured aphids using the same two aphid genotypes as the parasitism assays. For each replicate, we transferred 25, 3^rd^–4^th^ instar alatoid nymphs to a 100×15 mm, sterile, polystyrene petri dish containing moistened filter paper and an excised soybean leaflet (variety S19R5; NK, Golden Valley, MN). The petiole of each leaflet was placed in moist florist foam to prevent leaflet desiccation. To measure aphid exposure to fungal conidia, a glass cover slip was attached to each leaflet to allow for enumeration of conidia after aphid exposure to cultures.

We initiated a total of 20 replicates for each aphid isoline pair, 10 each from the infected and cured isolines. We used actively sporulating *P. neoaphidis* cultures to inoculate aphids. Subcultures used in the assays had been established 30–40 days prior to use and were only used after sporulation became evident (i.e., when conidia became visible on culture lids). All fungal cultures originated from the same *P. neoaphidis* isolate, which had been initially isolated from an infected, field-collected pea aphid (*Acyrthosiphon pisum*). The field collected isolate was used to infect soybean aphids in the laboratory, after which, the fungus was recovered from a single infected soybean aphid. The resulting isolate was periodically passed through and recovered from single soybean aphid individuals prior to use in the assays. Such periodic infection and recovery was necessary to maintain culture pathogenicity. Cultures used to infect soybean aphids in these assays originated from a single culture recovered from an infected soybean aphid immediately prior to assay initiation. The *P. neoaphidis* isolate has been deposited in the USDA, Agricultural Research Service's Collection of Entomopathogenic Fungal Cultures (ARSEF 11663).

Fungal cultures were inverted over each soybean aphid replicate. After 2 h, the fungal cultures and coverslip were removed from each replicate, and the dishes were sealed with parafilm to maintain the humidity required for fungal disease initiation. Each cover slip was stained with aceto-orcein stain, and examined at 200× magnification. Spores had been deposited on all, indicating that all replicates were exposed to fungal conidia. We then counted spores in 10 randomly chosen fields of view per replicate, and calculated mean spore number per field as an estimate of fungal exposure.

We examined the aphids once per day over the next 5 days. Dead or apparently infected aphids were removed from the experimental dish and transferred to a 50 mm tissue culture dish containing 1% water agar to induce sporulation. If sporulation occurred, the aphid was considered to be infected. We confirmed fungal species identity for two aphids exhibiting successful sporulation on each of the 5 days that assays were monitored. Conidia were stained with aceto-orcein stain and species identity was confirmed via spore morphology at 200× magnification [66].

We calculated the proportion of aphids infected per replicate, and used Pearson's correlation coefficient to determine whether this value was significantly associated with fungal exposure per replicate. We observed substantial variation in both variables, but they were not strongly correlated (*R* = 0.067, *P* = 0.72), so we proceeded to compare fungal infection between treatments without including fungal exposure as a covariate. We arcsine square-root transformed the proportion of aphids infected by *P. neoaphidis*, and performed t-tests (IBM SPSS v20) to determine whether this proportion differed as a function of *Arsenophonus* presence/absence in either aphid isoline.
